# Caregiver Strategies to Sensory Features for Children With Autism and Developmental Disabilities

**DOI:** 10.3389/fpsyg.2022.905154

**Published:** 2022-07-22

**Authors:** Lauren M. Little, Karla Ausderau, Ashley Freuler, John Sideris, Grace T. Baranek

**Affiliations:** ^1^College of Health Sciences, Rush University, Chicago, IL, United States; ^2^Department of Kinesiology, University of Wisconsin-Madison, Madison, WI, United States; ^3^Chapel Hill-Carrboro City Schools, Carrboro, NC, United States; ^4^Chan Division of Occupational Science and Occupational Therapy, University of Southern California, Los Angeles, CA, United States

**Keywords:** autism spectrum disorders, participation, sensory processing, caregivers, routines

## Abstract

**Background:**

Caregivers of children with autism spectrum disorders (ASD) and developmental disabilities (DD) implement myriad strategies to support their children during daily activities and routines, which are laden with sensory stimuli. Children’s sensory features are often characterized by three patterns of response (i.e., hyperresponsiveness, hyporesponsiveness, sensory seeking), and little is known about how caregivers’ strategies differ among these patterns. Therefore, we used a mixed methods analysis to examine the complex interplay between children’s sensory response patterns, child characteristics (diagnosis, chronological age, mental age), and caregiver strategies. Specifically, we examined how children’s sensory response pattern scores were associated with caregiver strategies within sensory response pattern and at the item level. Lastly, we described the differential strategies implemented by caregivers of children with ASD and DD by sensory response pattern.

**Materials and Methods:**

Participants included children with ASD (*n* = 77) and DD (*n* = 40) aged 2–10 years. Caregivers completed the Sensory Experiences Questionnaire-2.1. A convergent parallel mixed methods approach was used to analyze data.

**Results:**

Children’s sensory response pattern scores were significantly, positively associated with caregiver strategies within each sensory pattern (hyperresponsiveness, hyporesponsiveness, seeking); however, child mental age, and chronological age were not significantly related to the rate of caregiver strategies across patterns. While caregivers of children with ASD reported using more strategies, child diagnosis did not moderate the association between child sensory response pattern scores and the rate of caregiver strategies used. Item analysis demonstrated specific child behaviors in response to sensory stimuli that elicited high rates of strategies among caregivers. Qualitative analysis revealed distinct themes characterized caregiver strategies within each sensory pattern for children with ASD and DD.

**Conclusion:**

Our findings demonstrated specificity of caregiver strategies to children’s sensory response patterns in the context of families’ everyday lives, which were not contingent on child diagnosis, mental age, or chronological age, thereby highlighting universal qualities of caregiving for young children who experience varying levels of sensory challenges. Targeted intervention approaches may differentially incorporate types of strategies based on sensory response patterns to more optimally facilitate children’s activity participation.

## Introduction

Among families of children with developmental disabilities (DD) and autism spectrum disorders (ASD), research suggests that children’s sensory features, or behavioral responses to sensory stimuli, influence families’ daily activities and routines (Bagby et al., 2012; Dunn et al., 2016; Pfeiffer et al., 2017). Studies show that caregivers implement myriad strategies to support child engagement in everyday activities and these strategies are often matched to specific child characteristics such as communication skills (e.g., Bernheimer and Weisner, 2007; Kirby et al., 2017; Pfeiffer et al., 2017) and self-care abilities (Kellegrew, 2000). However, the ways in which caregivers use strategies in response to children’s specific sensory features has been less researched. For example, caregivers may dampen the sensory stimuli of certain activities (e.g., turning down sound, using a softer toothbrush) or amplify the sensory input of other activities (e.g., using brighter lighting, offering a trampoline for more intense movement experience). Given that sensory features are highly prevalent among both children with ASD and DD (Baranek et al., 2006; Tomchek and Dunn, 2007; Dunn, 2007; Ausderau K. K. et al., 2014; Kirby et al., 2022), the investigation of specific caregiver strategies in response to these child characteristics is needed.

Children’s sensory features are commonly grouped into three patterns of response: hyperresponsiveness (HYPER), hyporesponsiveness (HYPO), and sensory seeking (SEEK) (Ausderau K. et al., 2014; Dunn et al., 2016; Baranek et al., 2019). HYPER is characterized by an exaggerated response to and/or aversion to sensory stimuli (i.e., distress during grooming) (Schoen et al., 2008; Ausderau K. et al., 2014). HYPO is described by a lack of or under response to sensory stimuli (i.e., lack of reactivity to pain) (Ben-Sasson et al., 2007; Watson et al., 2011). SEEK has been described as fascination with or craving sensory stimuli (e.g., fascination with the visual appearance of water) (Boyd et al., 2010; Kirby et al., 2016).

Evidence points to the ways that caregivers consider the ways that sensory qualities of daily activities interact with the sensory processing preferences and aversions of children with disabilities. Qualitative research has illuminated the role that sensory features play in the everyday activities of children with ASD, pointing to how caregivers implement specific strategies, such as changes to daily routines, to mitigate their children’s responses to sensory stimuli (Rodger and Umaibalan, 2011; Schaaf et al., 2011; Bagby et al., 2012). Moreover, caregivers of preschool aged children with ASD reported that their children’s sensory features contributed to their lack of participation, in addition to the parents’ lessened provision of opportunities for participation (LaVesser and Berg, 2011). Bagby et al. (2012) found that among families of children with ASD, children’s unusual sensory features impact what a family chooses to do or not do, how the family prepares for occupations, and the extent to which experiences, meaning, and feelings are shared during occupations. In another study, Schaaf et al. (2011) reported that families of children with ASD expressed the need to be flexible with daily activities, especially outside of the home, due to the children’s unpredictable responses to sensory stimuli. Pfeiffer et al. (2017), in a qualitative study on how caregivers perceived the influence of sensory environments on child participation, found that caregivers of young children with ASD implemented specific strategies to promote child participation based on whether the activity was essential or not.

While studies have illuminated how caregivers consider children’s sensory features when planning and engaging in everyday activities, research has not uncovered specific strategies in which caregivers differentially employ in response to children’s sensory response patterns. Instead, caregiver strategies to support child engagement in activities may be contingent on child characteristics, such as chronological age or cognitive abilities. While not specific to sensory issues, one early study by Kasari et al. (1988) showed that children’s mental age was related to caregivers’ implementation of strategies, such as parents of children with intellectual disabilities provided increased gestures to facilitate dyadic engagement while those of children with ASD provided more physical supports. In another study, Dumas et al. (2003) reported that parents of children with acquired brain injuries (ABI) used routine, repetition, and consistency; supports and modeling; and curriculum and environmental modifications to promote child participation. Additionally, parents of children with ABI identified that they used following strategies across home, community, and school contexts: creating opportunities, teaching skills, and supporting child cognitive and behavioral regulation (Bedell et al., 2005). Caregivers of children with ASD have reported that they not only implement various strategies to promote child participation in the context of sensory challenges, but these strategies may differ in the context of home- vs. community-based activities (Kirby et al., 2017). Clearly, caregivers of children with varying developmental conditions implement strategies based on child characteristics and more research is needed to understand these differential strategies based on child sensory response patterns.

This research addresses a number of gaps in the literature on the intersection of child sensory processing and caregiver strategies. First, while research has established that caregivers of children with ASD and DD implement strategies in response to children’s responses to sensory information (e.g., Weisner et al., 2005; Bagby et al., 2012; Kirby et al., 2017; Pfeiffer et al., 2017), there is little information about how the intensity of children’s sensory preferences and aversions influences the amount or types of strategies used by caregivers. That is, is it unclear if children with intense behavioral responses to specific sensory stimuli result in a higher utilization of specific strategies among caregivers. For example, some caregivers may implement strategies when children demonstrate mild responses to sensory stimuli, while others may have a higher threshold for child responses such that they do not use strategies when children’s sensory features are more extreme.

Second, it is unclear if child diagnosis differentially contributes to the ways that caregivers use strategies to optimize child participation in daily activities. Previous studies of caregiver strategies have used relatively diagnostic homogenous samples of children (e.g., Schiavone et al., 2018), contributing to a limited understanding of potential commonalities of caregiver strategies. It may be that there are specific child characteristics associated with ASD (e.g., differences in social interaction, communication, repetitive behavior) that influence caregiver strategies. Conversely, there may be universal qualities of caregiving for young children with developmental delays, regardless of diagnosis, that emerge when children show responses to sensory information. Therefore, we tested the influence of diagnosis on the association between rate of caregiver strategies and children’s sensory response pattern scores.

Third, it is unclear if types of caregiver strategies differ by sensory response pattern. It is likely that caregivers use particular strategies in response to children’s hyperresponsiveness vs. hyporesponsiveness. However, research has largely taken a qualitative approach and focused on general strategies to children’s sensory features (e.g., changing the routine, limiting community outings) instead of parsing out strategies that correspond with specific child behaviors that reflect each sensory response pattern in specific contexts. Our research questions included:

1. Does the intensity of children’s sensory response patterns predict the rate of caregiver strategies, as moderated by child diagnostic group (ASD, DD), chronological age, or mental age?
2. Do caregivers of children with ASD vs. DD differ in the extent to which they implement strategies in response to specific child behaviors?
3. How do caregivers describe the strategies that they implement specific to each sensory response pattern?

## Materials and Methods

### Design

A convergent parallel design was used to evaluate our research questions (Creswell and Clark, 2007). Within mixed methods, decisions are made related to timing of data collection and mixing (relating the two data sets) of each approach (Creswell and Clark, 2007). For the current study, we collected all data at the same time point using one instrument (Sensory Experiences Questionnaire 2.1 [SEQ.2.1] Baranek, 1999) that has both a qualitative (descriptive text) and quantitative (frequency ratings) component. We first examined the quantitative ratings associated with item frequencies of children’s behavioral responses across the three sensory response patterns as well as the amount of parent strategies endorsed. Then, we examined the qualitative data to identify *specific types* of caregiver strategies that the quantitative data did not address. To integrate findings, we compared how the quantitative and qualitative findings converged and diverged. Our purpose was to not only identify differences in the associations between sensory pattern and *overall frequency* of caregiver strategies, but also gain insight into the nature of the specific strategies in which caregivers implemented and how these strategy types were differentially characterized within each of the three sensory response patterns.

### Participants

Participants were caregivers of children aged 2–10 years with a diagnosis of either ASD (*n* = 77) or DD (*n* = 40) (see Table 1). Data were collected as part of a larger grant-funded study, and caregivers gave written informed consent as approved by the University’s Institutional Review Board. Children included in the ASD group had been diagnosed by a licensed psychologist and had met criteria on the Autism Diagnostic Observation Schedule (Lord et al., 1999). Children in the DD group had a diagnosis associated with intellectual disability (e.g., Down syndrome) or of non-specific origin (e.g., speech language disorder). Exclusionary criteria for all groups included: significant visual or hearing impairments, seizure activity, and genetic conditions that are often comorbid with ASD, such as fragile X syndrome. In the current analysis, we excluded *n* = 2 children with ASD and *n* = 2 children with DD due to incomplete data (e.g., lack of cognitive assessment).

**TABLE 1:** Participant demographics.

Diagnostic group	N	% male	Race/Ethnicity n (%)	CA Mean (SD) Range (months)	MA Mean (SD) Range (months)
ASD	77	78.8	African American = 9 (11.7) White = 56 (79.2) More than 1 race = 7 (9.1) Hispanic = 4 (5.2)	51.58 (16.30) 20–84	34.37 (20.80) 4–69
DD	40	55.0	African American = 3 (7.5) Asian = 1 (2.5) White = 32 (80.0) More than 1 race = 4 (10.0) Hispanic = 2 (5.0)	48.18 (23.06) 20–118	32.2 (17.25) 8–69

### Measures

#### Sensory Experiences Questionnaire Version 2.1 (SEQ-2.1)

This study used the SEQ-2.1 (Baranek, 1999) a 43-item caregiver report instrument designed to evaluate everyday sensory experiences in children. The SEQ-2.1 measures sensory features across the three sensory response patterns (HYPO, HYPER, SEEK) in both social and non-social contexts, and across all modalities. Previous research demonstrated that the SEQ shows discriminant validity between children with DD, ASD, and typical development for those aged 6 months to 6 years (Baranek et al., 2006). Additionally, the SEQ shows high internal consistency (α = 0.80), and test-retest reliability (ICC = 0.92) (Little et al., 2011).

The items on the SEQ 2.1 are each divided into three parts: (a), (b), and (c). Questions in part (a) ask the caregiver to rate the frequency of occurrence of a child’s sensory experience, based on a 5-point Likert scale (1 = almost never to 5 = almost always). Thus, part (a) of the questionnaire provides a quantitative metric of the child’s behavioral response to each item; scores are summed to derive total scores across each of the three sensory response patterns: HYPER, HYPO, and SEEK. Part (b) of each SEQ 2.1 item asks the caregiver to choose (yes/no) if he/she attempts to change the child’s behavior (i.e., uses a strategy in response to child behaviors associated with specific sensory experience). From part (b), the number of “yes” scores is summed to calculate the proportion of strategies implemented for each sensory response pattern. Part (c) requests the caregiver to qualitatively describe the specific types of strategies used in these situations. Part (c) is an open ended question that allows caregivers to convey qualitative information about the strategies that they employ in response to children’s sensory features; such strategies may be those that were modeled in therapies or as part of the caregiver’s personal choices based on their problem solving in context with their child. An example of parts a-c of an item on the SEQ 2.1 includes: (a) How often does your child refuse new foods? (b) Do you attempt to change this behavior; and (c) If yes, please describe. For the current mixed methods analysis, we utilized quantitative data from parts (a) and (b) of the SEQ-2.1, and qualitative, descriptive text responses from caregivers from part (c) of the questionnaire.

#### Child Chronological and Mental Age

Child chronological age was calculated from the child’s birthdate to the completion of the SEQ 2.1. A variety of measures were used to test the child’s cognitive functioning, including the Bayley II- Mental Developmental Index (Bayley, 1993), Mullen Scales of Early Learning (Mullen, 1995), and/or the Leiter-R (Roid and Miller, 1997), depending upon the child’s age and ability level. Mental age (MA) was derived directly from cognitive assessments or extrapolated from standard scores. All cognitive assessments were administered within 4 weeks within completion of the SEQ-2.1.

## Quantitative Data Analysis

SPSS 17.0 was used to analyze quantitative data. Children’s sensory scores were obtained from the SEQ 2.1 (part a) for each item and summed within the HYPER, HYPO, and SEEK scales. Similarly, caregiver strategy scores were obtained and summed from the SEQ 2.1 (part b) for each scale. First, we used *t*-tests to examine potential chronological and mental age differences between children with DD and ASD in the sample. Second, we used a series of regression analyses to examine: (a) the influence of children’s scores on HYPER scales on the reported frequency of parent HYPER strategies; (b) the influence of children’s scores on HYPO scales on the reported frequency of parent HYPO strategies; and (c) the influence of children’s scores on SEEK scales on the reported frequency of parent SEEK strategies. In each model, we also tested the moderating effects of diagnosis (ASD, DD), chronological age, and mental age.

Lastly, we used chi-square tests to examine differences between caregivers that endorsed “Yes” vs. “No” for Part (b) on each of the SEQ 2.1 items (i.e., if caregiver used strategy in an attempt to change the child’s behavior). This analysis allowed us to understand if strategy use significantly differed by item and by diagnosis (ASD, DD).

## Quantitative Results

The two diagnostic groups (ASD, DD) did not significantly differ on MA (*p* = 0.555) or CA (*p* = 0.356). Given that these child variables may influence caregiver strategy use, however, we tested the influence of MA and CA in regression models. Regression results are shown in Table 2. Overall, MA and CA did not demonstrate significant main or moderating effects on the association between sensory response pattern scores and rate of strategy use within each sensory response pattern, and were therefore removed from subsequent models. In other words, child chronological and mental age were not significant predictors of caregiver reported rate of strategy use within each sensory response pattern, showing that the significant predictors of caregiver reported strategies were the child’s sensory response pattern scores and diagnostic category.

**TABLE 2:** Strategy use as predicted by sensory response pattern scores.

	t	B	*p*	Adjusted *R* Squared
**HYPO strategy use**				
Hyporesponsiveness score	6.825	0.159	0.000	0.399
Diagnosis	3.289	0.112	0.001	
**HYPER strategy use**				
Hyperresponsiveness score	7.287	0.155	0.000	0.426
Diagnosis	2.520	0.062	0.013	
**SEEK strategy use**				
Seeking score	2.889	0.068	0.005	0.123
Diagnosis	2.189	0.074	0.031	

Next, we analyzed if the rate caregiver strategies (i.e., endorsements for attempts to change child behavior) differed at the item level on the SEQ 2.1 (see Table 3). Ten items on the SEQ 2.1 showed there were significant differences in caregiver endorsement of strategies by diagnostic group, with caregivers of children with ASD reporting increased attempts to change children’s behavior (e.g., avoid looking at face, flap arms, or hands) with specific strategies. Four items (i.e., *Refusal of new foods*, *Ignore being called by name*, *Shows distress during grooming*, and *Putting objects in mouth*) were endorsed highly (>50%) in both groups as situations where caregivers invoked various strategies in response to child sensory behaviors. Strategies for *Refusal of new foods* was the highest endorsed (85.7% of caregivers with ASD, 62.5% of those with DD), followed by strategies for *Ignore being called by name* (84.4% of caregivers with ASD, 62.5% of those with DD). Child sensory behaviors with the lowest reported endorsement of caregiver strategies included *Dislike being tickled* (3.9% of caregivers with ASD, 0% of those with DD) and *Ignore loud noises* (1.3% of caregivers with ASD, 2% of those with DD).

**TABLE 3:** Item level results.

SEQ 2.1 item	Reported strategy use ASD, DD n (%)	*X*^2^ (df, N)	*p*-value
**Do you attempt to change your child’s following behaviors…**			
Refuse new foods*^a^* (HY)	66 (85.7), 25 (62.5)	8.208 (1, 117)**	0.004
Ignore called by name*^a^* (HO)	65 (84.4), 25 (62.5)	7.123 (1, 117)**	0.008
Avoid looking at face*^a^* (HY)	58 (75.3), 7 (17.5)	35.649 (1, 117)**	0.001
Show distress during grooming*^a^* (HY)	55 (71.4), 25 (62.5)	0.971 (1, 117)	0.325
Put objects in mouth*^a^* (SK)	48 (62.3), 26 (65.0)	0.080 (1, 117)	0.777
Startle to loud sounds (HY)	33 (42.9), 8 (20.0)	6.042 (1, 117)*	0.014
Ignore something new entering room (HO)	37 (48.1), 3 (7.5)	19.240 (1, 117)**	0.001
Ignore being tapped (HO)	31 (40.3), 8 (20.0)	4.862 (1, 117)*	0.027
Like to jump, rock, spin (SK)	23 (29.9), 5 (12.5)	4.363 (1, 117)*	0.037
Slow to notice new objects (HO)	23 (29.9), 2 (5.0)	9.691 (1, 117)**	0.002
Stare at objects (SK)	22 (28.6), 2 (5.0)	8.970 (1, 117)**	0.003
Flap arms or hands (SK)	19 (24.7), 1 (2.5)	9.134 (1, 117)**	0.003
Avoid touching certain textures (HY)	28 (36.4), 9 (22.5)	2.340 (1, 117)	0.126
Distress at loud conversation (HY)	19 (24.7), 6 (15.0)	0.226 (1, 117)	0.226
React negatively when touched (HY)	15 (19.5), 3 (7.5)	2.903 (1, 117)	0.088
Disturbed by too much light (HY)	14 (18.2), 5 (12.5)	0.625 (1, 117)	0.429
Dislikes cuddling (HY)	14 (18.2), 3 (7.5)	2.419 (1, 117)	0.120
Dislike being in water (HY)	13 (16.9), 4 (10.0)	1.004 (1, 117)	0.316
Slow to react to pain (HO)	11 (14.3), 3 (7.5)	1.151 (1, 117)	0.283
Seek out rough-housing (SK)	12 (15.6), 4 (10.0)	0.695 (1, 117)	0.404
Interested in way people smell (SK)	6 (7.8), 1 (2.5)	1.311 (1, 117)	0.252
Uneasy on a swing (HY)	5 (6.5), 3 (7.5)	0.042 (1, 117)	0.838
Notice sounds in environment (HY)	4 (5.2), 2 (5.0)	0.964 (1, 117)	0.964
Smell objects or toys, change behavior (SK)	4 (5.2), 2 (5.0)	0.002 (1, 117)	0.964
Dislike being tickled (HY)	3 (3.9), 0	1.599 (1, 117)	0.206
Ignore loud noises (HO)	1 (1.3), 2 (5.0)	0.230 (1, 117)	0.230

^a^>50% of participants in one diagnostic group reported strategy use; *<0.01; **<0.05; HR, hyperresponsivness; HO, hyporesponsiveness; SK, sensory seeking.

### Qualitative Data Analysis

Given the non-significant interactions between diagnostic group and sensory response pattern, we analyzed the qualitative data with data from caregivers of children with ASD and DD combined. The quantitative analysis suggested the number of caregiver strategies were contingent on the level of sensory response pattern scores rather than diagnostic group. Therefore, thematic analysis was conducted by combining diagnostic groups and examining themes within sensory response patterns (hyperresponsiveness, hyporesponsiveness, sensory seeking).

ATLAS.ti 6.2 (Muhr, 2004) was used for data management and to support analysis of qualitative responses (i.e., text) from the SEQ-2.1 (part c) describing the nature of the types of strategies parents implemented. Each pattern was analyzed separately using thematic analysis as outlined by Braun and Clarke (2006) to characterize caregiver strategies within each sensory pattern. This iterative process of analysis began with individual team members reviewing all of the qualitative responses within sensory pattern while generating preliminary codes and initial ideas about the data. Through team discussion, the codes were then collated and further refined within each sensory response pattern and ideas surrounding broader themes were discussed. The codes and themes were again reviewed and discussed by the team. The iterative and reflexive process continued until codes and themes were agreed upon by all team members. The broader themes that best represented the data for each sensory response pattern were then further refined, defined, and named. Within each sensory response pattern, clear themes emerged that characterized caregiver strategies as follows.

## Qualitative Findings

### Hyperresponsiveness Sensory Pattern

#### Step by Step

Caregivers reported strategies that reflected a graded approach to reducing their children’s aversive responses to everyday activities, and these strategies were meant to facilitate increased participation in the desired activity over time. Caregivers reported attempts to try to slowly introduce or break up the activity into small components to encourage participation. One caregiver reported, regarding her child’s avoidance of touching certain textures, “*I try a little bit more each day as long as he does not get upset.*” In response to a question about her child’s refusal of new foods, another parent indicated a graded approach to her child’s picky eating: “*Try to offer new foods, eat them and enjoy them in front of him, have peers eat the same food.*”

#### Remove and Avoid

In contrast, this theme described a process by which caregivers implemented strategies during everyday activities aimed at removing the child from the aversive experience, extinguishing the aversive stimuli, or planning an activity to ensure the stimuli would not be present. Children’s aversions to loud sounds elicited caregiver responses such as: “*Extinguish the source of the sound or try to move her away from it*” and “*Adjust volume, remove him from environment or give him some space further from it.*” Another caregiver reported, “*Try to have him go to a quieter place in the house if the vacuum is on.*” An example of a caregiver planning an activity to ensure the child would not be exposed to an aversive stimuli would be, “*[I] do not use the vacuum when he is home.*”

#### Whatever We Need to Do to Get Through It, Because It Has to Be Done

Caregivers reported that certain activities, regardless of their children’s aversive responses, are necessary aspects of everyday routines. Caregivers subsequently did whatever they could to get through these activities with their children, and attempted any strategy aimed at completing certain tasks. With regard to a child’s distress during grooming, one parent reported, “*Teeth brushing is the worst–we have been just fighting through it*.” Another caregiver reported that during grooming activities, “*Provide a lot of support; toys to hold onto during haircutting; Try to make teeth brushing as fun as possible; Brush his sister’s teeth at the same time so he can see her do it*.” Caregivers described a determination for completing the activity in the presence of the child’s strong aversive responses and providing support for the child when possible during completion.

#### Soothe and Comfort

Caregivers’ implemented strategies aimed at simply calming or comforting the child during or in anticipation of aversive reactions. In response to a question about children’s distress during loud conversations or singing, a caregiver reported, “*We just comfort and reassure him.*” Similarly, a caregiver expressed how they accommodate due to the child’s aversive reactions to loud voices: “*Is frightened by men with loud voices (like his uncle). [He] cries when he is near. I try to tell him it’s okay*.”

### Hyporesponsiveness Sensory Pattern

#### Engagement

When children demonstrated HYPO behaviors (i.e., a lack of/delayed response to sensory stimuli during everyday activities), the strategies that caregivers reported emerged as one overarching theme: *Engagement.* Caregivers described strategies to promote their children’s involvement in various everyday activities and these efforts reflected a desire to increase their children’s interactions with individuals. Two subthemes described strategies within this broader theme, including: *Persistence Using Multiple Strategies to Engage* and *Explanation and Encouragement Surrounding Engagement.*

Caregivers of children with ASD and DD reported that they utilized a number of different strategies in an attempt to gain a response from their child, sometimes increasingly salient in nature, which were labeled as *Persistence Using Multiple Strategies to Engage*. In response to a question regarding the child’s ignoring his name being called, one caregiver reported that she “*speaks[s] louder; claps hands; go get him; make eye contact.*” In response to the child appearing to ignore someone new entering the room, a caregiver reported, “*I always let him know who’s coming; introduce him; ask him to say “Hi”.*” Strategies within this theme reflected a sequence of strategies that caregivers used specifically to elicit engagement from the child.

In contrast, the second subtheme reflected caregivers’ efforts to encourage engagement through explanation and support. For instance, in response to a child’s ignoring name call, a caregiver reported, “*We remind him to look and listen.*” Another caregiver related, “*Explain that she needs to respond when called.*” One caregiver explained, when her child ignores or tunes out loud noises, “*I point out that which has happened and why.*” Caregivers used strategies that provide the child with knowledge and encouragement primarily through verbal input.

### Sensory Seeking Pattern

#### Do Not Do It

This theme reflected caregivers’ efforts to directly limit or constrain children’s sensory seeking behaviors. The strategies reported in SEEK conveyed that such child behaviors are not tolerated, and caregivers reported efforts to stop the behaviors after the child had begun to engage in them. For example, in response to her child mouthing non-food items, a caregiver reported, “*Make him spit them out and explain it’s not safe to put anything not food in his mouth.*” In response to a similar question, another caregiver related, “*[I] tell him “NO”.*”

#### Redirect and Replace

As opposed to efforts to directly eliminate sensory seeking behaviors, this theme reflected caregivers’ strategies aimed at either altering the child’s behavior into something socially acceptable or substituting the behavior for another way to meet the child’s sensory needs. At times, caregivers would encourage a different behavior or direct the child to a different activity. With regard to a child’s hand flapping, one caregiver reported that she attempted to redirect by “*diverting [his] attention, put something in [his] hands.*” Another caregiver reported that in response to her child’s flapping, she will “*try to redirect him.*”

#### It’s Okay…Sometimes

The last theme reflected how caregivers attempted to meet their child’s sensory seeking behaviors within specific limits, and how some children may be allowed to engage in sensory seeking behaviors in certain contexts or at certain times. For instance, in response to children’s jumping, one caregiver reported that she stopped the behavior “*Only if it’s out in public or he could get hurt–I ask him to stop–he usually does.*” Another caregiver allowed her child to jump in a specific environment, the child’s room, and constructed that setting specifically for sensory seeking behaviors: “*His room has been emptied out of all furniture and replaced with crash pad, foamies, and bop toys.*”

## Discussion

This study used a mixed methods approach to examine caregiver strategies based on children’s sensory response patterns. Novel findings from this study showed that regardless of a child’s diagnosis (ASD or DD), higher levels of children’s sensory behaviors were associated with significantly increased use of caregiver strategies to address the sensory experiences. Interestingly, we found that the association between sensory response pattern and caregiver strategy use was not significantly influenced by mental or chronological age in this sample of children with ASD and DD. This finding suggests that there may be universal qualities about children’s responses to sensory stimuli that elicit caregiver involvement regardless of the children’s overall child cognitive or developmental level among those with developmental conditions.

Quantitative results suggested that the level of children’s hyperresponsiveness, hyporesponsiveness, and sensory seeking, but not necessarily their diagnostic group, were significant predictors of the amount of caregiver strategies used during everyday activities. Item level analyses revealed that caregivers of children with ASD and DD implemented similar rates of strategies in response to specific child behaviors, aligned with hyperresponsiveness (e.g., refusal of new foods) and hyporesponsiveness (e.g., ignoring name being called). Contrastingly, there may be behaviors that align with the diagnostic features of ASD that caregivers attempt to increasingly change (e.g., avoid looking at face), which contributed to the significant differences in rate of item level strategies among diagnostic groups. Clearly, some behaviors associated with sensory response patterns are more acceptable to caregivers, as they reported low use of strategies (e.g., avoiding certain textures) to intervene in these situations. Also, certain strategies may be easier to implement during daily routines (e.g., not purchasing clothing of certain textures), and caregivers may learn over time to structure children’s activities and environments to match their children’s sensory preferences and aversions.

Findings pointed to the ways that caregivers implemented different types of strategies based on the three sensory response patterns. While some research suggests that parents use strategies in response to children’s overall sensory processing differences (Kirby et al., 2016), results from this study suggest that the *types* of strategies used within each sensory response pattern are distinct. Thus, strategies used to support children when they display hyperresponsiveness in daily activities are qualitatively different from those strategies used for hyporesponsiveness or sensory seeking behaviors.

Thematic analysis further revealed the type and range of strategies caregivers may employ to help children cope with hyperresponsiveness to sensory stimuli during daily activities. Previous research has demonstrated that specifically among children with ASD, children’s aversive responses to sensory stimuli during meal times and grooming present particular challenges for caregivers (Kientz and Dunn, 1997; Tomchek and Dunn, 2007). Item analysis findings in the current study aligned with these previous findings, and showed that children’s refusal of new foods and distress during grooming are areas in which caregivers highly endorse as having to implement strategies. Further, our analysis extended previous research by suggesting adaptive strategies are implemented similarly across diagnoses to help children cope with sensory-laden situations that trigger hyperresponsiveness. In some situations, caregivers may eliminate (i.e., *Remove and Avoid*) elements of activities that elicit severe discomfort. In other situations, caregivers employ strategies that systematically expose their children (i.e., *Step by Step*) and gradually desensitize them over time so they can more fully participate in these activities.

While previous research has focused on hyperresponsiveness among children with ASD and DD in the context of daily activities, fewer studies have focused on hyporesponsiveness. Harrop et al. (2018) reported that caregivers of minimally verbal children with ASD most frequently reporting prompting, followed by redirection strategies. Aligned with these findings, qualitative and quantitative results from this study revealed the value caregivers place on their child’s active involvement in daily activities and social interactions in the presence of hyporesponsiveness. The child’s degree of hyporesponsiveness significantly predicted the amount of strategies that were implemented by caregivers. *Engagement*, a salient theme from the qualitative data, characterized the ways in which caregivers of children with ASD and DD responded to their children’s hyporesponsiveness, implementing two different intensities of strategies (verbal and/or multi) to initially facilitate the child’s response, as well as explanation and encouragement to maintain their child’s participation in everyday activities.

Qualitative analysis revealed that caregivers may respond to children’s sensory seeking behaviors in a number of ways, including allowing or encouraging behaviors, encouraging replacement behaviors, or attempting to eliminate such child behaviors. Studies have characterized children’s sensory seeking as a fascination or intense interest in the sensory elements of activities (Dunn, 2007; Boyd et al., 2010; Kirby et al., 2017), however, previous research on the ways in which sensory seeking impacts families’ activities and the strategies that they use is limited. Thematic analysis suggested that caregivers may be implementing fewer strategies in response to their children’s sensory seeking behaviors if they interpret the child’s behaviors as pleasurable or serving a regulatory purpose. If caregivers perceive a child’s sensory seeking behaviors as pleasurable or regulatory, or perhaps as having a shared meaning with the caregiver (Bagby et al., 2012), they may be less likely to implement strategies to limit such child behaviors. Previous research (Spitzer, 2003) reported that a mother perceived her child’s sensory seeking behaviors as a way in which to engage with her non-verbal child, and emerging evidence from autistic self-advocates suggests that sensory seeking behaviors serve specific purposes (e.g., Ekblad and Pfuhl, 2017). Conversely, the theme *Do not Do It* suggests that select caregivers may not tolerate certain sensory seeking behaviors for various reasons, and implement strategies in an attempt to eliminate these behaviors. For example, some caregivers may perceive that a particular sensory seeking behavior poses danger to a child (e.g., mouthing non-food items) or others may find a behavior (e.g., flapping, spinning in public contexts) to be inappropriate or disruptive to their family’s social situation. Although caregiver interpretations of the function of their child’s sensory seeking behaviors were beyond the current investigation, future research may further illuminate how such perceptions influence caregivers’ strategies.

### Limitations and Future Directions

There are several limitations of this work. First, the SEQ 2.1 was the only instrument used in this study and utilized a written parent-report format; future studies could incorporate observational methods as well as in-depth interviews to corroborate or expand upon these findings. Second, we described the amounts and types of strategies used for each sensory response pattern, but could not evaluate the relative effectiveness of these strategies, which could be expanded upon in future studies. Evidence has shown that the three sensory response patterns often co-occur (Ben-Sasson et al., 2007; Ausderau et al., 2016) and that varying sensory related subgroups show differential outcomes (Ausderau et al., 2016; Tomchek et al., 2018), thus future studies could assess the degree to which caregiver strategies differ across subgroups of children with mixed patterns of response, and how these strategies may moderate outcomes. Future mixed methods research may further explore how caregivers’ attitudes, beliefs, and other psychological characteristics (e.g., levels of stress) influence the types of strategies used in the context of their children’s sensory response patterns, and their relative effectiveness to support children’s engagement in daily activities and overall participation. Lastly, the current research does not delineate how caregivers learned the strategies that they reported to employ in response to children’s sensory features. Future studies may investigate the extent to which enrollment in different types of therapies (e.g., occupational, physical, speech therapies) influence the frequency and type of strategies in which caregivers use.

## Data Availability Statement

The data supporting the results and analyses presented in this article will be made available by the authors, upon reasonable request.

## Ethics Statement

The studies involving human participants were reviewed and approved by University of North Carolina at Chapel Hill. The patients/participants provided their written informed consent to participate in this study.

## Author Contributions

GB: data collection. All authors: study conception, design, analysis and interpretation of results, draft manuscript preparation, reviewed the results, and approved the final version of the manuscript.

## Conflict of Interest

GB is the author of the Sensory Experiences Questionnaire version 2.1 but receives no royalties since it is freely available. The remaining authors declare that the research was conducted in the absence of any commercial or financial relationships that could be construed as a potential conflict of interest.

## Publisher’s Note

All claims expressed in this article are solely those of the authors and do not necessarily represent those of their affiliated organizations, or those of the publisher, the editors and the reviewers. Any product that may be evaluated in this article, or claim that may be made by its manufacturer, is not guaranteed or endorsed by the publisher.
